# Antibacterial Effect of Potassium Tetraborate Tetrahydrate against Soft Rot Disease Agent *Pectobacterium carotovorum* in Tomato

**DOI:** 10.3389/fmicb.2017.01728

**Published:** 2017-09-12

**Authors:** Firas A. Ahmed, Mohammad Arif, Anne M. Alvarez

**Affiliations:** Department of Plant and Environmental Protection Sciences, University of Hawaii at Manoa, Honolulu HI, United States

**Keywords:** *Pectobacterium carotovorum*, post-harvest disease, tomato, PMA-qPCR cell viability, flow cytometry

## Abstract

Soft rot caused by *Pectobacterium carotovorum* is one of most common bacterial diseases occurring in fruits and vegetables worldwide, yet consumer-acceptable options for post-harvest disease management are still insufficient. We evaluated the effect of potassium tetraborate tetrahydrate (B_4_K_2_O_7_.4H_2_O) (PTB) on the growth of *P. carotovorum* using strain BA17 as a representative of high virulence. Complete inhibition of bacterial growth was achieved by treatment with PTB at 100 mM both at pH 9.2 and after adjustment to pH 7.0. Bactericidal activity was quantified and validated by counting fluorescently labeled live and dead bacterial cells using flow cytometry, and reconfirmed using qPCR with high-affinity photoreactive DNA binding dye propidium monoazide (PMA). The results of flow cytometry, qPCR, and culturing confirmed that bacterial cells were killed following exposure to PTB at 100 mM. Bacterial cell membranes were damaged following a 5-min treatment and extrusion of cytoplasmic material from bacterial cells was observed using electronic transmission microscopy. Soft rot incidence on inoculated tomato fruit was significantly reduced by dipping infected fruits in PTB at 100 mM for 5 min and no lesions developed following a 10-min treatment. PTB does not pose a hazard to human health and is an effective alternative to other bactericides and antibiotics for controlling soft rot disease of tomato caused by *P. carotovorum.*

## Introduction

Tomatoes are an important economic crop worldwide and soft rot disease caused by *Pectobacterium carotovorum* leads to significant post-harvest losses. Soft rot is a predominant progressive decay characterized by tissue maceration, such that the entire fruit is damaged (Bartz and Wei, 2002; Gardan et al., 2003; Charkowski, 2007). Bacterial infections occur in the field, transit, packing, and/or during storage. Quantitative losses caused by soft rot are greater than any other bacterial disease (Bhat et al., 2010). The bacteria are not able to penetrate the surface of tissue directly but rather enter through wounds and natural openings. The bacteria then multiply in the intercellular spaces, where they produce pectolytic enzymes and degrade cell middle lamellae leading to softening of infected tissue (Yang et al., 1992). Currently, there are few effective ways to decontaminate infected tomato fruit that do not pose concerns to human health; thus, disease management essentially depends on cultural practices such as avoiding over-irrigation, maintaining proper harvesting, handling and packing practices, cleaning and disinfestations of harvesting equipment, and use proper storage conditions (Gnanamanickam, 2006; Jones et al., 2014).

Recent studies have shown the efficiency of inorganic salts, such as aluminum chloride, sodium thiosulfate, and sodium benzoate to control post-harvest diseases such as soft rot of potato (Yaganza et al., 2014), gray mold of grapevines and tomato (Jeandet et al., 2000; Wu et al., 2015), decay of melons (Aharoni et al., 1997), and citrus green mold (Smilanick et al., 1999). Potassium tetraborate tetrahydrate (B_4_K_2_O_7_.4H_2_O) (PTB) is one of the inorganic salts used for preservation wood worldwide (Freeman et al., 2009). Some studies demonstrated the effectiveness of PTB application to control common post-harvest fungal diseases, such as gray mold caused by *Botrytis cinerea* in table grapes (Qin et al., 2010), anthracnose caused by *Colletotrichum gloeosporioides* in mango (Shi et al., 2011, 2012). PTB has been used in industry and agriculture as safe compounds for control of many fungi and insects (Qin et al., 2007). However, there is no documentation on the use of PTB to control post-harvest diseases caused by bacteria. Flow cytometry is a rapid sensitive method for quantifying of live and dead cells in suspension (O’Brien-Simpson et al., 2016; Massicotte et al., 2017). Propidium monoazide (PMA) is high-affinity photoreactive DNA binding dye that has recently been used to distinguish intact from membrane- damage of bacterial cells and other microorganisms. PMA only penetrates into dead bacterial cells with damaged membrane but not live cells with the intact membrane (Nocker et al., 2006). PMA Combined with qPCR was successfully used to quantify the viability of bacterial cells (Bae and Wuertz, 2009; Kim and Ko, 2012). The objective of this study was to investigate the effectiveness of PTB for reducing soft rot of tomato caused by *P. carotovorum*.

## Materials and Methods

### Bacterial Strain

The bacterial strain used for these studies was *P. carotovorum* BA17, which was the most virulent strain isolated in Hawaii. Pathogenicity was confirmed on three types of tomato including, common market, cherry and grape tomato. Several bacteriological tests including oxidation/fermentation (OF), production of catalase, degradation of sodium polypectate, KOH test and hydrolysis of esculin and starch were conducted on each strain. Presumptive identifications were confirmed by 16S rDNA sequence analysis (Ahmed et al., 2017). All strains were maintained in freezers at -80°C for further testing.

### Determination of Antibacterial Activity and Minimum Bactericidal Concentration of PTB *In Vitro* Conditions

The antibacterial activity of PTB (Sigma–Aldrich, St. Louis, MO, United States) was assessed for *P. carotovorum* using a paper disk diffusion method (Jorgensen and Turnidge, 2015). One ml of an overnight broth culture of bacteria was diluted to obtain an inoculum of 10^8^ CFU/ml using a spectrophotometer (600 nm). A 50 μl aliquot was spread evenly on solid agar plates containing Luria media using sterile swabs. Sterile 13-mm paper disks were saturated with PTB in increasing concentrations ranging from 10 to 120 mM. The first tests were run at pH 9.2 (with no pH adjustment) and a second set was run after adjustment to pH 7.0 following neutralization with 0.1 N HCl. Plates were divided into four quarters, and one paper disk was placed in each quadrant. Paper disks saturated with sterile water were used as controls. Plates were incubated at 28°C for 24 h, and the inhibition zone area was measured. For determination of minimum bactericidal concentration (MBC), tubes containing Luria broth and PTB at concentrations ranging from 10 to 100 mM were inoculated with 20 μl aliquots of a *P. carotovorum* suspension that contained 10^8^ CFU/ml. Tubes were placed in the incubator shaker for 16 h at 28°C, 140 rpm. Two controls were used: (i) inoculated Luria broth lacking PTB, and (ii) non-inoculated broth. After incubation, Aliquots (20 μl) of resuspended media were spotted onto Luria agar plates divided into four quadrants. Plates were incubated at 28°C for 24 h. Plates were checked daily over the next 7 days to determine whether growth occurred. The lowest concentration of PTB that killed the *P. carotovorum* was recorded as MBC. The experiments were performed with four replicates and repeated three times. To confirm that the effect was bactericidal and not bacteriostatic, the experiment above was repeated except that broth cultures were centrifuged, the pellet containing bacterial cells were washed three times with sterile saline, and resuspended in Luria broth lacking PTB. Aliquots were again spotted onto Luria agar plates and incubated as above. This experiment was repeated twice.

### Validation of Antimicrobial Activity of PTB Using Flow Cytometry Analyses

Flow cytometry was performed to validate the bactericidal activity of PTB using viability dyes, Syto9 (membrane permeable) and propidium iodide (PI, membrane impermeable) (O’Brien-Simpson et al., 2016). Control samples of live (measured by colony plate counts) and dead cells (killed by 70% isopropanol) were first established as controls for flow cytometry runs before determining the numbers of live and dead cells in samples treated with PTB. Liquid NB 24 h old cultures of *P. carotovorum* were treated with different concentrations of PTB (50-, 60-, 70-, 80-, 90-, and 100-mM) for 16 h at 28°C with shaking at 140 rpm. The bacterial suspensions were centrifuged, and the pellet was washed twice with 0.85% NaCl. Bacterial cells were stained with the LIVE/DEAD *Bac*Light kit components of Syto9 and propidium iodide (Thermo Fisher, Grand Island, NY, United States) following the protocol provided by the manufacturer. Samples were analyzed with a Beckman-Coulter EPICS XL flow cytometry instrument (FlowJo, Ashland, OR, United States) using 15 mW 488 nm excitation (argon ion laser). The fluorescence signals from the Syto9-stained cells (LIVE) were collected (green 525 nm BP filter), along with PI-stained cells (DEAD) (red 610 nm BP filter). Forward and side light scatter signals were measured. Two-parameter histograms of log PI fluorescence vs. log Syto9 fluorescence were analyzed in FlowJo software, which is designed to distinguish live and dead cells and to calculate the percentage of each cell type. Each experiment was conducted with four replicates, and the test was repeated three times.

### Validation of Antimicrobial Activity of PTB Using qPCR-Based Method with Propidium Monoazide (PMA) to Distinguish Viable from Non-viable Bacteria Cells

A PMA assay was conducted using the BLU-V Viability PMA kit (Qiagen, Valencia, CA, United States) following the manufacturer’s instructions. A suspension of *P. carotovorum* containing 10^8^ CFU/ml was treated with 100 mM PTB in nutrient broth for 16 h at 28°C with shaking 140 rpm. Four control samples were prepared: live and dead bacterial cells each with and without PMA. As a control, bacterial cells were killed by heating for 10 min at 70°C in a thermomixer. Treated and non-treated bacterial suspensions were centrifuged for 5 min at 13,000 *× g.* A 10 μl volume of 2.5 mM of PMA reagent was added to the cell pellet containing 500 μl of EB buffer. After incubation for 10 min at room temperature in the dark, samples were exposed to light for 20 min using a 650-W halogen light source (Kobayashi et al., 2009). The samples were laid on the ice at a 45 angle and 20 cm in front of the light source. After photoactivation of PMA, the bacterial suspension was centrifuged at 7,600 rpm for 10 min, and DNA was extracted.

### DNA Extraction and SYBR Green Real-time qPCR

A DNeasy Blood & Tissue Kit (Qiagen) was used to isolate DNA from PMA-treated and non-treated *P. carotovorum* cultures following the manufacturer’s protocol. Primer set PEC-F (5′-GTGCAAGCGTTAATCGGAATG-3′) and PEC-R (5′-CTCTACAAGACTCTAGCCTGTCAGT TT-3′) targeting the 16S rRNA gene (Pritchard et al., 2013) was used for SYBR Green qPCR-based specific detection of *P. carotovorum;* primers were synthesized by Integrated DNA Technologies (IDT, Coralville, IA, United States). SYBR Green qPCR assays were performed in a 25 μl reaction mixture containing 12.5 μl SsoAdvance Universal Supermix (Bio-Rad, Hercules, CA, United States), 8.5 μl of ultrapure water (Thermo Fisher Scientific, Grand Island, NY, United States), and 0.2 μM of each forward and reverse primer. SYBR Green qPCR conditions were 30 s at 95°C followed by 30 cycles of 10 s at 95°C and 30 s at 60°C using CFX96 Real-Time PCR Detection System (Bio-Rad). Three replicates were used for each reaction and water was used as a non-template control. Cycle threshold was set manually, and Bio-Rad CFX Manager 3.1 software was used to analyze the data.

### Transmission Electron Microscopy (TEM)

The possible damage to bacterial membranes treated with and without PTB was assessed using TEM following the method of Al-Adham et al. (2000) with slight modification. An overnight culture *P. carotovorum* (BA17) was grown on NB supplemented with or without 100 mM PTB for 16 h at 28°C with shaking 140 rpm. One-ml aliquots were centrifuged at 4000 × *g* for 10 min at room temperature and washed two times with sterile distilled water. The pelleted cells were fixed with 4% glutaraldehyde in 0.1 M sodium cacodylate buffer, pH 7.2, held at room temperature overnight and washed in 0.1 M cacodylate buffer 2x for 10 min, followed by post-fixation with 1% OsO_4_ in 0.1 M cacodylate buffer for 1 h. Cells were dehydrated in a graded ethanol series (30, 50, 70, 85, 95, and 100%), substituted with propylene oxide, and embedded in LX112 epoxy resin and placed in a 60°C oven for 24 h to polymerize the resin. Ultrathin (60–80 nm) sections made on a resin-fixed pellet using RMC PowerTome Ultramicrotome and double stained with uranyl acetate and lead citrate. Sections were examined utilizing a Hitachi HT7700 TEM (Hitachi, Tokyo, Japan) at 100 kV and photographed with an AMT XR-41B 2kx and 2k CCD camera.

### *In Vivo* Effects of PTB on Soft Rot Disease Symptoms on Tomato Fruit

#### Protective Action of PTB

A method using potassium tetraborate tetrahydrate was used to assess the efficacy of PTB for protection the tomato fruit from soft rot caused by *P. carotovorum*. Tomato fruits that were uniform in size and color, free from wounds and rot were selected. The fruits were washed with tap water; surface sterilized by dipping in 1% sodium hypochlorite solution for 10 min, rinsed by immersing in two changes of sterile distilled water, and dried in ambient air. A wound 1 mm diameter in 4 mm deep was made on each fruit using a pipette tip. Wounded fruits were immersed for 10 min in the different PTB concentrations (0-, 90-, 100-, and 120- mM) for 1-, 2-, 5- and 10- min, and then dried in ambient air. Each wound was inoculated with a 10-μl aliquot containing 1 × 10^8^ CFU/ml of *P. carotovorum* (Chen and Zhu, 2011). Bacterial suspensions were prepared from 24 h-old cultures grown on Luria agar (Difco, Sparks, MD, United States). Fruits were placed onto wet paper towels in a plastic container and incubated for 3 days at 24°C. Disease severity was assessed by measuring lesion diameter on each fruit. Each treatment contains five fruits. Lesion diameter was the mean of the five fruits in each replicate. The experiment was repeated twice.

#### Curative Action of PTB

A curative assay was conducted using the procedures used for the protective assay except that the wounded fruit were immersed in the borate solution for 12 h after inoculation (Yaganza et al., 2014). Each treatment was replicated by five fruits and the test was repeated twice.

### Statistical Analysis

The experiments were set up as complete randomized design (CRD) with four replications. Data were analyzed using SAS 9.2 V (SAS Institute, Inc., Cary, NC, United States) and means were compared by Duncan’s multiple range tests. Differences at *p* < 0.05 were considered significant. The experiments were repeated two to three times.

## Results

### Molecular Identification

A PCR product was obtained for all bacterial strains with expected size 1.4 kb. An NCBI BLAST that sequences of BA12 and BA15 were matched > 98% with *Pectobacterium* spp. For BA17, the similarity was ≥ 100% to *P. carotovorum* (Ahmed et al., 2017).

### Antimicrobial Activity of PTB at pH 7.0 and pH 9.2

**FIGURE 1:**
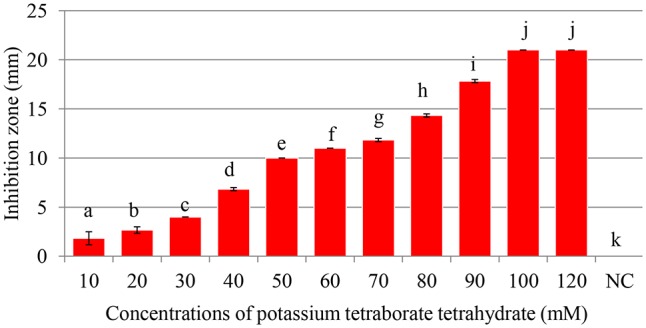
Antibacterial activity of of potassium tetraborate tetrahydrate (PTB) at pH 9.2. Maximum inhibition of *Pectobacterium carotovorum* occurred at 100 mM. Vertical bars show mean values and standard error (±SE). Means with different letters are statistically different according to Duncan’s multiple range test (*p* < 0.05).

**FIGURE 2:**
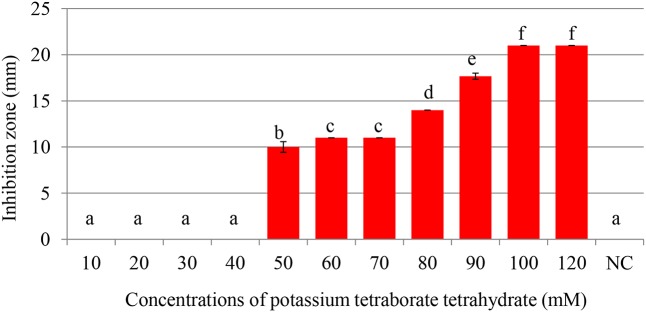
Inhibition of *Pectobacterium carotovorum* by increasing concentrations of potassium tetraborate tetrahydrate (PTB) at pH 7.0. Bacterial growth was partially inhibited at 50 mM; maximum inhibition zones were produced at 100 mM PTB. Vertical bars show mean values and standard error (±SE). Means with different letters are statistically different according to Duncan’s multiple range test (*p* < 0.05).

Maximum inhibition of *P. carotovorum* occurred at 100 mM. The inhibition zone increased with the increase of PTB concentrations (*p* < 0.05). A 100 mM, the inhibition zone was 22 mm compared with control (water only) 0.00 mm regardless of the pH (**Figures 1**, **2**). There was not different in inhibition zone at 100 and 120 mM PTB (*p* > 0.05). These results confirm that while the high pH (pH 9.5) accounts for some of the bactericidal activity of PTB, high pH is not the sole explanation for its bactericidal action.

### Minimum Bactericidal Concentration (MBC)

**FIGURE 3:**
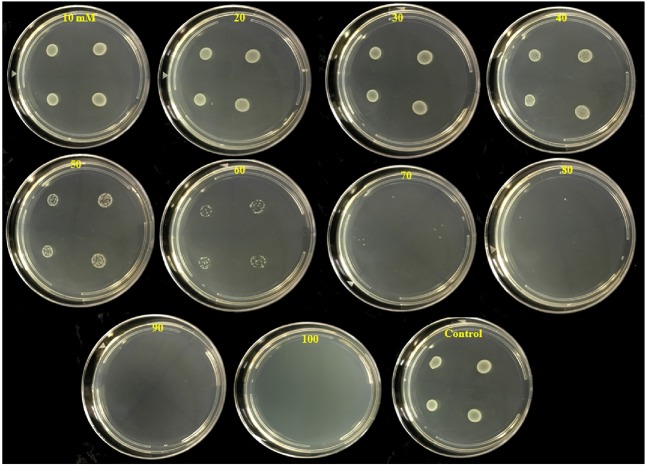
Inhibition of colony development of *P. carotovorum* by increasing concentrations of potassium tetraborate tetrahydrate (PTB). Aliquots (10 μl) containing 10^7^ CFU of *P. carotovorum* were deposited at four sites on a series of Petri plates which contained different concentrations ranging from 10 to 100 mM PTB. Two colonies developed on plates containing 80 mM, one colony was visible on the plates containing 90 mM and no colonies developed on plates containing 100 mM PTB.

The number of colonies that grew on test plates decreased as the PTB concentrations in the medium increased (**Figure 3**). No bacterial colonies appeared on plates containing 100 mM PTB after 24 h incubation at 28°C, and there was no regrowth on these plates after 7 days. Furthermore, after washing pelleted cells three times before plating onto Luria agar, no colonies grew from tubes containing 100 mM potassium tetraborate tetrahydrate, and this was established as the MBC.

### Flow Cytometric Analysis of Bactericidal Activity of PTB

**FIGURE 4:**
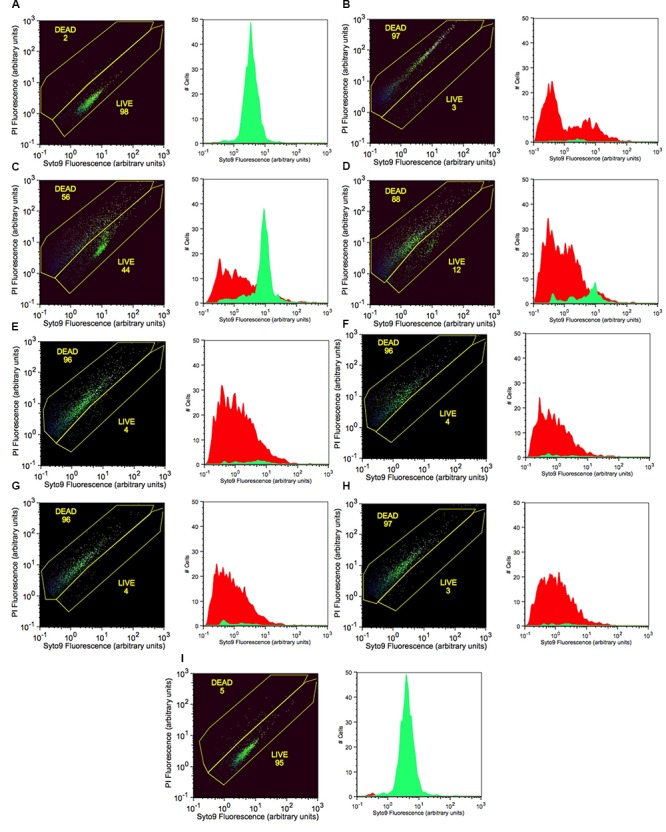
Increasing concentrations of potassium tetraborate tetrahydrate (PTB) increases bacterial membrane permeability to propidium iodide as shown by flow cytometry with Syto9 and PI staining. **(A)** live cells (non-treated; calibration standard); **(B)** dead cells (killed with 70% isopropanol; calibration standard). Bacterial cells treated with PTB at increasing millimolar concentrations **(C)** 50; **(D)** 60; **(E)** 70; **(F)** 80; **(G)** 90; **(H)** 100; **(I)** non-treated bacteria (control). The X-axis indicates the intensity of Syto9 green fluorescence and the Y-axis indicates the intensity of PI red fluorescence (arbitrary units: a). flow cytometry data are represented as the percentage of live and dead cells (black images) with the fluorescence intensity of Syto9 and PI dyes. Graphs show the images present the cell counts of live (green) and dead (red) cells.

Live cells with intact cell membranes were distinguished from dead isopropanol-treated bacterial cells as shown on flow cytometry dot blots and histograms (**Figure 4**). Live cells of *P. carotovorum* stained with Syto9 dye and clustered in the lower gate (98% of the data points) whereas 2% of the cells stained with propidium iodide and clustered in the upper gate (**Figure 4A**). Conversely, with the dead-cell-control, 97% of the data points were in the upper gate whereas 3% of the data points were in the lower gate (**Figure 4B**). Following treatment with 50 mM PTB 56% of the cells were dead and 44% were live (**Figure 4C**). The percentage of dead cells in the upper gate continued to increase as the PTB concentrations increased (**Figures 4D–H**). The non-treated cells stained with Syto9 dye and 95% clustered in the lower gate and gave a histogram similar to the live cell-control.

### Real-time qPCR with PMA Treatment for Analysis of Bactericidal Activity of PTB

**FIGURE 5:**
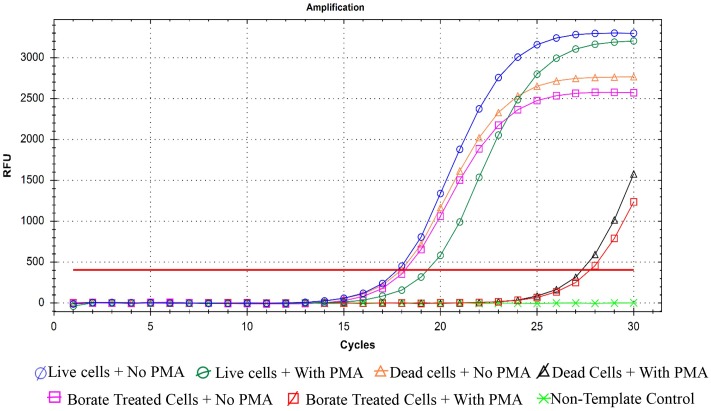
Quantification real-time PCR for *P. carotovorum* treated with 100 mM potassium tetraborate tetrahydrate (PTB) with and without propidium monoazide (PMA). Data are averages of three separate experiments.

Quantitative PCR was performed as an efficient additional method for confirming bactericidal activity of PTB at 100 mM. When PMA was absent from bacterial suspensions, qPCR did not distinguish between live and dead cells (**Figure 5**). Addition of PMA to live bacterial suspensions did not change the Ct values because PMA cannot penetrate membranes of intact cells and had no effect on qPCR. In contrast, when PMA penetrates the membranes of dead bacterial cells, it binds to the DNA and delays amplification, which results in higher Ct values for the sample. Both the heat- and PTB-treated cells had significantly higher Ct values (25) than live cells (Ct value, 15). The results obtained by PMA-qPCR coincided with results of flow cytometry and culture plate assays confirming complete kill of bacterial cells with 100 mM PTB.

### Effect of PTB on Ultrastructure of Bacterial Cell Analyzed by TEM

**FIGURE 6:**
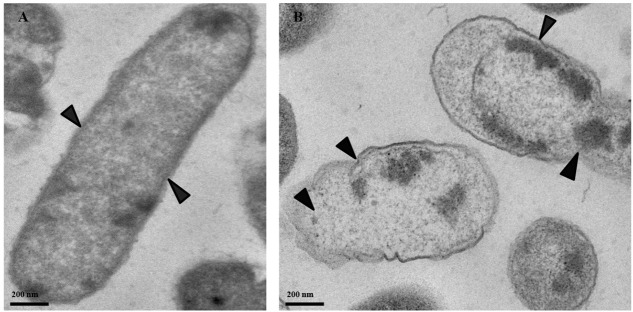
Micrographs of *P. carotovorum* cells examined with transmission electron microscopy (TEM). Bacteria were incubated in nutrient broth medium (NB) without potassium tetraborate tetrahydrate (PTB) **(A)** or with 100 mM PTB **(B)**. In the absence of borate, the cytoplasm and cell membranes were intact and appeared normal (**A**; black arrows). Following treatment with 100 mM PTB the cell membranes were degraded, the cytoplasm exuded from damaged areas and cell structure was abnormal (**B**; black arrows).

Transmission electronic microscopy was used to visualize morphological changes in *P. cartovorum* cells treated with 100 mM PTB. Cell membranes and cytoplasm were normal showing no noticeable changes in morphological structure in the absence of PTB (**Figure 6A**). The cells in the untreated control sample of *P. carotovorum* showed intact cell membranes with no deformation or dense cytoplasm. In contrast, bacterial cells grown in nutrient broth supplemented with 100 mM PTB showed distinct signs of deterioration (**Figure 6B**). The cell membrane was distorted and the cytoplasm exuded from membranes of all the cells observed.

### Effect of PTB on Soft Rot Disease of Tomato Fruit

**FIGURE 7:**
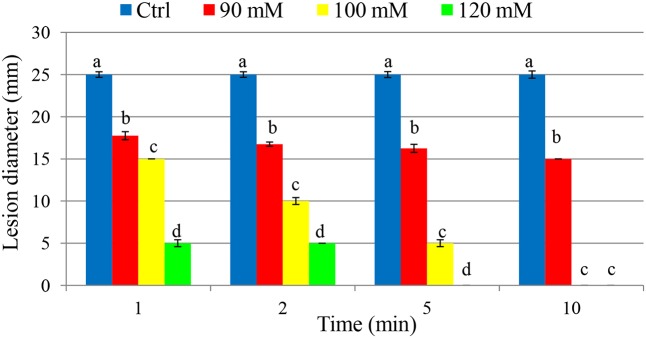
Effect of different potassium tetraborate tetrahydrate (PTB) concentrations at different immersion times on disease severity in tomato fruit following inoculation with *P. carotovorum*. The mean lesion diameter was measured after the treated fruit were stored at 28°C for 7 days. Mean values showing different letters are statistically different according to Duncan’s multiple range test (*p* < 0.05).

As a curative treatment, PTB was not effective in reducing soft rot disease (*p* > 0.05). As a preventive treatment, however, PTB applications at 100 and 120 mM completely inhibited lesion development (**Figure 7** and Supplementary Figure 1). Fruits dipped into PTB (100 and 120 mM) for 5 and 10 min prior to inoculation differend in lesion diameters after 7 days incubation at 23°C compared with the control or treatments at lower rates (*p* < 0.05) (**Figure 7**). These results were consistent with data from MBC, flow cytometry, and qPCR tests. The results indicate that PTB reduced post-harvest soft rot of tomato fruit when fruits were immersed in 100 mM PTB for 5 min. Lesion development was completely inhibited following emersion for 10 min.

## Discussion

Boron is an essential microelement for plant growth and enhancement of fruit quality. In tomato adequate levels of boron are needed to avoid ruptures in the cuticle (Strong and Robert, 2001; Huang and Snapp, 2009). Recently, several studies have demonstrated that PTB can be used as an antifungal compound for control of post-harvest diseases (Qin et al., 2010; Thomidis and Exadaktylou, 2010; Shi et al., 2011, 2012). Other studies provided evidence that boron (Kartal et al., 2004) and PTB (Reeb, 1997) reduced wood damage by termites and fungi. Our study was the first to evaluate the potential of PTB for post-harvest management of soft rot bacterial disease caused by *P. carotovorum*. PTB at 100 mM completely inhibited the growth of *P. carotovorum*, both at its normally high pH (9.5) and after neutralizing to pH 7.0. Salt solutions of PTB, potassium carbonate, sodium bicarbonate and sodium carbonate also exhibited antifungal and antibacterial effects independently of pH (Nigro et al., 2006; Qin et al., 2010). Thus, the antibacterial activity of PTB was not due solely to disruption of cell membranes at high pH. The inhibitory effect of boron (as borax and boric acid) on *Escherichia coli*, *Staphylococcus aureus*, *Pseudomonas aeruginosa*, and *Actinetobacter septicus* was similar with and without adjusting the pH value (Yilmaz, 2012).

Several possible mechanisms for the inhibitory effect of borate include binding of borate ions to chemical energy transporters such as ATP, NAD, and NADH leading to impaired protein synthesis, disruption of mitochondria and prevention of cell division (Kim et al., 2003; Reid et al., 2004). Excessively, high salt concentrations disrupt osmoregulatory processes and reduce bacterial growth. High alkalinity can denature protease on the cell surface, change the cytoplasmic pH and disrupt DNA (Gould and Russell, 1991). The water-ionizing capacity and the lipophilicity of the inorganic salts components play an important role in the inhibition of bacterial growth (Yaganza et al., 2009). In addition to water-ionizing capacity, borate salt is possible to possess the feature of hydrophobic nature that would enable the borate salt to interact with lipid components of the bacterial cell membrane. This interaction leads to dysfunction of the bacteria and results in growth inhibition.

The bactericidal activity of PTB against *P. carotovorum*, was confirmed in our study by flow cytometry using a protocol to assess bactericidal activity (O’Brien-Simpson et al., 2016). Increased PI staining of *P. carotovorum* cells treated with PTB indicates cell membrane disruption resulting in a bactericidal rather than a bacteriostatic effect. The relationship between the borate concentration and the percentage of dead cells was generally linear. The analysis by flow cytometry confirmed results observed in the cultural antibacterial assays. No bacterial colonies developed following a 10-min treatment with PTB at 100 mM or higher. As a further validation, we used quantitative PCR with propidium monoazide, which is a DNA binding agent that can only penetrate cells with severely damaged membranes. PMA has been used in combination with quantitative PCR to differentiate intact from dead bacterial cells (Kobayashi et al., 2009; Desfossés-Foucault et al., 2012). The Ct value of borate-treated bacteria was similar to the Ct value of the dead-cell control, indicating that PTB destroyed cell membranes permitting the PMA to mask DNA and make the maximum primer binding sites unavailable. These results confirm results of the cultural and flow cytometry tests. Transmission electron microscopy (TEM) showed degradation of bacterial cell membranes, exudation of cytoplasm and abnormal cell structure, indicating that treated cells were damaged by PTB. Antifungal action of PTB also was associated with damage to *Colletotrichum gloeosporioides* (Shi et al., 2011, 2012) and *Botrytis cinerea* (Qin et al., 2010). *Pectobacterium atrosepticum* cells were rapidly damaged and killed by exposure to sodium metabisulfite and aluminum chloride salts (Yaganza et al., 2004).

As a preventative treatment, PTB applications showed a significant reduction in the development of soft rot disease of tomato fruit. However, it had no curative action, most likely because bacterial multiplication cannot be arrested once the bacteria enter wounded fruit. PTB is a promising bactericidal agent for controlling soft rot disease caused by *P. carotovorum* and presents low risk to human health (Çelikezen et al., 2014). In addition, application of PTB is inexpensive and can be applied to tomato fruit before shipping to the market.

## Conclusion

PTB salt at 100 mM was clearly bactericidal against *P. carotovorum* and its bactericidal activity was confirmed by TEM, flow cytometry and qPCR. Preventive 1–5-min dip applications of potassium tetraborate at 100 mM significantly reduced lesion diameters in fruits inoculated with *P. carotovorum.* No lesions developed following a 10-min dip treatment. PTB can be a safe and cost-effective alternative for preventing soft rot disease in post-harvest tomato fruit.

## Author Contributions

FA and AA designed the study, FA performed the experiments and wrote the manuscript, FA and MA conducted qPCR. MA and AA revised the manuscript and provided ideas and support for the final submission. All authors reviewed the manuscript.

## Conflict of Interest Statement

The authors declare that the research was conducted in the absence of any commercial or financial relationships that could be construed as a potential conflict of interest.
