# Supplementary material for: Antibacterial Effect of Potassium Tetraborate Tetrahydrate against Soft Rot Disease Agent Pectobacterium carotovorum in Tomato
Source: Front Microbiol. 2017 Sep 12;8:1728. doi: 10.3389/fmicb.2017.01728 (PMC5601058; doi:10.3389/fmicb.2017.01728)
Supplement: Supplementary file 1 [file Image_1.pdf]

Supplementary Material

Antibacterial effect of potassium tetraborate tetrahydrate against soft rot disease agent *Pectobacterium carotovorum* in tomato

Firas A. Ahmed, Mohammad Arif, Anne M. Alvarez[*]

*** Correspondence: Anne M. Alvarez**: alvarez@hawaii.edu

1 Supplemental Figures and Tables

1.1 Supplemental Figure

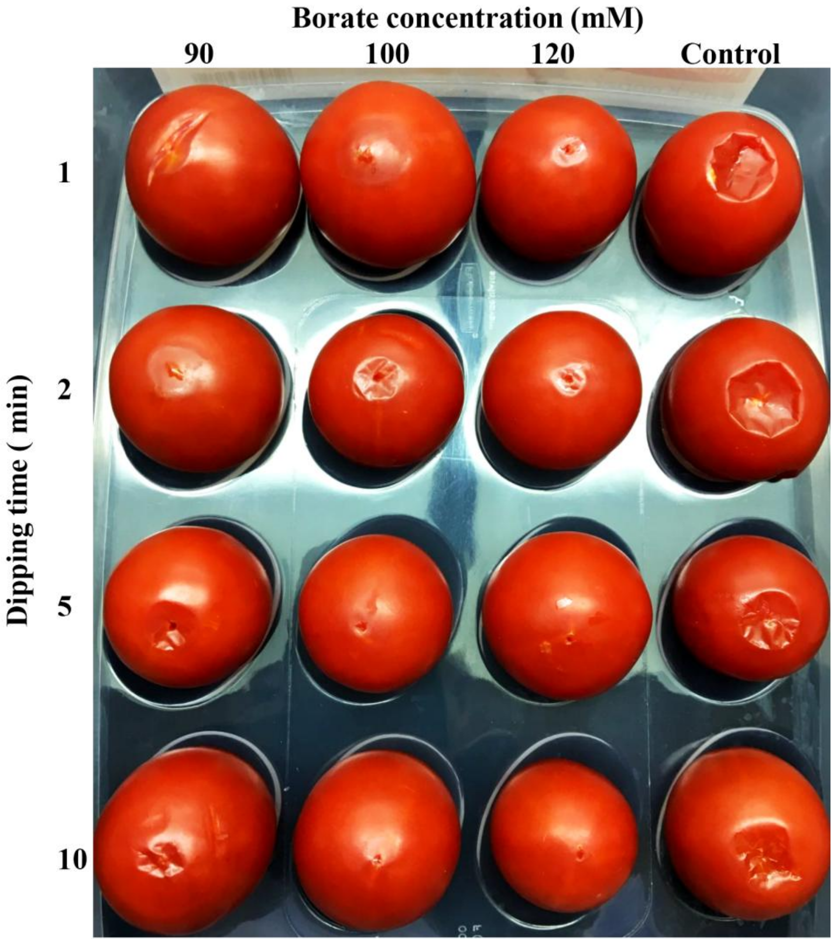

Supplemental Figure 1. Photograph showing efficacy of potassium tetraborate tetrahydrate (PTB) on disease severity on tomato fruit with varing concentrations and immersion times. Fruit were treated 10 min prior to inculation with *Pectobacterium carotovorum*. Inoculated fruit were stored for 7 days at 28˚C then evaluated. The photograph was taken seven days after inoculation.
